# The Psychometric Properties and Clinical Usefulness of the Difficulties in Emotion Regulation Scale in Forensic Settings

**DOI:** 10.1002/jclp.70193

**Published:** 2026-07-23

**Authors:** Adam Meddeb, Märta Wallinius, Johan Berlin, Natalie Laporte, Malin Hildebrand Karlén, Claudia Mazzeschi, Elisa Delvecchio, Patrizia Velotti, Carlo Garofalo

**Affiliations:** ^1^ Department of Clinical Sciences Lund Lund University Lund Sweden; ^2^ Center of Ethics, Law and Mental Health, Institute of Neuroscience and Physiology, The Sahlgrenska Academy University of Gothenburg Gothenburg Sweden; ^3^ Department of Research Regional Forensic Psychiatric Clinic Växjö Sweden; ^4^ Department for Forensic Psychiatry The National Board of Forensic Medicine Gothenburg Sweden; ^5^ Department of Psychology University of Gothenburg Gothenburg Sweden; ^6^ Department of Philosophy, Social Sciences and Education University of Perugia Perugia Italy; ^7^ Department of Dynamic and Clinical Psychology, and Health Studies Sapienza University of Rome Rome Italy

**Keywords:** assessment, emotion regulation, psychometric, reliability, validity

## Abstract

**Objective:**

The Difficulties in Emotion Regulation Scale (DERS) is a widely used measure of emotion dysregulation across clinical contexts. However, its psychometric properties and clinical utility have not been thoroughly examined in forensic populations. This study evaluated the original 36‐item DERS and two short forms, the DERS‐16 and the DERS‐8, in two independent forensic samples from two different countries: a forensic psychiatric inpatient sample (*N* = 88) and a prison sample (*N* = 389).

**Methods:**

Confirmatory factor analysis was used to assess factor structure. Reliability was evaluated using Cronbach's *α* and McDonald's *ω*, and construct validity was examined through correlations with aggression, externalizing psychopathology, impulsivity, mindfulness, and alexithymia.

**Results:**

The original six‐factor model showed poor model fit, whereas data‐model fit improved for the DERS‐16 and DERS‐8. The DERS total and subscale scores correlated meaningfully with measures of aggression, externalizing pathology, impulsivity, mindfulness, and alexithymia. However, DERS scores were not significantly associated with habitual use of specific emotion regulation strategies.

**Conclusion:**

Findings suggest that the DERS‐16 and DERS‐8 may be preferable to the full 36‐item scale in forensic settings, offering promising psychometric properties while minimizing patient burden. Beyond psychometric adequacy, the results provide clinically relevant insights into the feasibility and practical utility of DERS in forensic mental health settings.

## 1 Introduction

Emotion regulation (ER) is a multidimensional construct that encompasses several processes through which individuals monitor, evaluate, and modify their emotional reactions (Thompson 1994). Difficulties in these processes are commonly referred to as emotion dysregulation and can be conceptualized as impairments in adaptive emotional responding (Gratz and Roemer 2004). A growing body of research has shown that emotion dysregulation is implicated in the development and etiology of a wide range of psychopathologies (Aldao et al. 2010; Dryman and Heimberg 2018). As such, emotion dysregulation has been conceptualized as a transdiagnostic factor across a wide spectrum of mental health issues, including anxiety disorders, personality disorders, substance use disorders, and depression (Aldao et al. 2016; Cludius et al. 2020; Dimaggio et al. 2017; Sloan et al. 2017).

Over the past two decades, research on ER has gained traction within forensic psychiatry where emotion dysregulation has been broadly linked to aggression and antisocial behavior (Garofalo et al. 2022; Roberton et al. 2014, 2015). Alongside the empirical evidence, extensive theoretical developments have been made where ER is increasingly being recognized as an important component of the overarching constructs of self‐control and self‐regulation (Billen et al. 2022; DeLisi and Vaughn 2014; Seto 2019; Tamir 2021). Following the surge of research on ER within forensic settings, meta‐analytical reviews are now corroborating the associations between emotion dysregulation and intimate partner violence (Maloney et al. 2023; Neilson et al. 2023), psychopathy (Velotti et al. 2024), substance abuse (Stellern et al. 2023), and recidivism (Seto et al. 2023). In light of the increased attention to emotion dysregulation in relation to antisocial behavior and its implications in the development and maintenance of psychopathology, access to psychometrically sound measures of emotion dysregulation is of great importance within forensic practice and research.

The Difficulties in Emotion Regulation Scale (DERS‐36; Gratz and Roemer 2004) is one of the most widely used measures of emotion dysregulation (Martínez‐Priego et al. 2024). It is by a notable margin the most commonly employed measure of emotion dysregulation within forensic settings (Meddeb et al. 2026). The questionnaire was developed with the aim of providing a comprehensive measure of difficulties in emotion regulation. Gratz and Roemer (2004) proposed a priori four broad dimensions of ER; (1) the awareness and understanding of emotions; (2) the acceptance of emotions; (3) the ability to adapt behavior in accordance with long‐term goals when under emotional pressure; and (4) the ability to use ER strategies to modulate the emotional experiences. However, the results from the original factor analysis resulted in a six‐factor correlated traits model. The DERS was thus constructed with six subscales, each composed of five to eight items. The Strategies subscale measures to what extent the respondent believes it is possible to effectively regulate emotions. The Nonacceptance subscale assesses the respondent's tendency to have negative secondary emotions in response to their own negative emotions. The Impulse subscale taps the respondent's ability to control behavior when emotionally distressed. The Goals subscale assesses difficulties engaging in goal‐directed behavior when emotionally distressed. The Awareness subscale measures the tendency to attend to and acknowledge emotional responses, and finally, the Clarity subscale measures the extent to which individuals can differentiate between their emotions. Conceptually, Gratz and Roemer (2004) provide a comprehensive framework of ER and define emotion dysregulation as the relative absence of any or several abilities as outlined in the DERS. As such, emotion dysregulation may vary in degree as well as in kind within the aforementioned framework.

There is substantial evidence in support of the criterion‐related validity of the DERS‐36 as it has been associated with various psychiatric difficulties including eating disorders (Monell et al. 2022; Nordgren et al. 2020), anxiety (Hallion et al. 2018; McVey et al. 2022), depression (Fowler et al. 2014; Hallion et al. 2018; McVey et al. 2022), and stress (Hallion et al. 2018). Further evidence supporting the validity of the DERS comes from findings of mean differences across groups where such differences would be theoretically expected. For instance, emotion dysregulation is theorized to lie at the core of borderline personality disorder (BPD; Linehan 1993) In line with such theoretical expectations, it has been found that individuals with BPD exhibit higher levels of emotion dysregulation as measured by the DERS‐36 (Miola et al. 2022). Moreover, BPD symptom severity can be detected using the DERS‐36 (Osborne et al. 2017). These findings highlight that variations in DERS scores may be meaningful both between and within patient groups. It should be noted that the Awareness subscale does not exhibit the same consistent association with measures of anxiety and depression compared to the remaining subscales in the DERS‐36 (McVey et al. 2022; Hallion et al. 2018). The divergent pattern of criterion‐based validity for the Awareness subscale has been noted extensively in the previous literature on the DERS (e.g., Bardeen et al. 2012; Hallion et al. 2018). Another noted concern regarding the DERS is whether the different subscales show distinct patterns of associations with external correlates or whether it may be more parsimonious to focus on overarching levels of emotion dysregulation (i.e., the DERS‐36 total score; John and Eng 2014). Most studies examining the criterion‐related validity of the DERS‐36 have primarily been conducted in community or in outpatient clinical samples, with substantially less research evaluating its clinical utility and psychometric properties in forensic settings.

In addition, the internal structure of the DERS has been widely investigated in both nonclinical (Bardeen et al. 2012; Giromini et al. 2012; Li et al. 2018) and clinical samples (Fowler et al. 2014; Hallion et al. 2018; McVey et al. 2022; Nordgren et al. 2020; Osborne et al. 2017; Sörman et al. 2022). The results from these studies have been inconclusive. Whilst the original factor structure has been replicated in some studies (e.g., Fowler et al. 2014), other studies have found a better fit for a five‐factor model excluding items from the Awareness subscale (e.g., Bardeen et al. 2012; Hallion et al. 2018; Sörman et al. 2022) as the factor was found to converge poorly with the remaining DERS subscales. One reason for the low intercorrelation between the Awareness factor and the other DERS factors might be due to a method effect, namely that all items in the Awareness subscale are reverse‐coded (Bardeen et al. 2012, 2016). Other alternative models that have been tested empirically include bifactor models, which have generally been found to produce better fit indices than first‐order factor models (Hallion et al. 2018; Nordgren et al. 2020; Xu et al. 2021). Second‐order factor models have also been examined and have not outperformed—in terms of fit indices—the original six‐factor correlated traits model (Bardeen et al. 2012; Nordgren et al. 2020). In a recent meta‐analytical confirmatory factor analysis (CFA), a total of 12 models were tested, with the authors concluding that a revised six‐factor correlated model based on 32 items had the strongest empirical support (Raimondi et al. 2024). It should be noted that the results from the cited meta‐analysis were based on samples largely composed of females (70%), reflecting a broader tendency in the DERS literature to rely on predominantly female samples. However, measurement invariance of the DERS‐36 has been investigated in relation to age and gender in a sample of adults with eating disorders with no evidence of measurement noninvariance being found (Nordgren et al. 2020). To our knowledge, no studies have directly examined the factor structure and validity of the DERS in forensic populations, which are composed predominantly of males.

In order to reduce the burden on research participants and patients in clinical settings, several short forms have been developed, including the 18‐item based DERS short form (DERS‐SF, Kaufman et al. 2016), DERS‐18 (Victor and Klonsky 2016), DERS‐16 (Bjureberg et al. 2016), and DERS‐8 (Penner et al. 2023). One of the more commonly used short forms—co‐developed with the authors of the original full‐length version—is the DERS‐16 (Bjureberg et al. 2016). An important advantage of this version over other short forms is that the reduced set of items was administered as a stand‐alone measure, rather than being extrapolated following administration of the full‐length version. The DERS‐16 excludes all items from the Awareness subscale but retains two or more items from the remaining five subscales. Of relevance to this study, DERS‐16 has been used in an outpatient sample of adults with psychosis (Lawlor et al. 2021) where a five‐factor model showed good model fit, excellent internal consistency and good convergent validity, as evidenced by moderate to strong associations with measures of psychological distress and experiential avoidance. Further support for the reliability and validity of the DERS‐16 comes from a study conducted in adults with emotional disorders (Hallion et al. 2018), in which the DERS‐16 was not inferior to the DERS‐36 in accounting for variance in symptoms of anxiety and stress. Notably, Hallion et al. (2018) found that subscale scores in the DERS‐16 contributed unique information in explaining clinical severity, even when controlling for the composite score. This finding indicates that clinically useful information may be obtained from specific subscales.

Considering the challenges associated with participant and patient recruitment in forensic settings (Pedersen et al. 2021), access to brief, validated, and reliable measures may be particularly important in this context. From this perspective, the DERS‐8 is an interesting candidate (Penner et al. 2023). The DERS‐8 excludes all items from the Clarity and Awareness subscales and does not provide subscale scores. All items are prefaced with the phrase “When I'm upset” which provides a more uniform context for the respondent. In the developmental study of the DERS‐8, respondents were recruited from a public university, with the final sample composed primarily of female participants (77%). Item response theory, together with content considerations, guided item selection. The unidimensional structure of the DERS‐8 was subsequently replicated in an adolescent sample recruited from a private psychiatric hospital and evidenced good reliability and convergent validity comparable to that of the DERS‐36 and other validated short forms. It should be noted, however, that respondents within the clinical sample that was used to replicate the factor structure of the DERS‐8 and further examine its validity were excluded if they had an IQ < 70 or were diagnosed with an autism spectrum disorder or a psychotic disorder. As such, it remains largely unknown how the DERS‐8 performs in forensic populations, which are predominantly male and characterized by a high prevalence of severe mental illness—particularly in forensic psychiatric settings—including psychotic disorders and severe neurodevelopmental disorders (Laporte et al. 2021).

### 1.1 Current Study

The utility and importance of assessing emotion dysregulation in forensic populations is underscored by extensive theoretical and empirical work linking such difficulties to aggressive behavior, recidivism, and externalizing psychopathology. Although the DERS has been extensively examined across a wide range of clinical and nonclinical populations, its psychometric performance has not been thoroughly evaluated in forensic populations. Prior research on the DERS has largely relied on predominantly female samples and has often excluded individuals with severe mental illness, limiting generalizability to individuals in prison and in forensic psychiatric care. Accordingly, the present study aims to address this gap in the literature by examining the psychometric properties of the DERS in two independent forensic samples: a forensic psychiatric sample and a prison sample—populations that remain underrepresented in the literature on the DERS.

Beyond evaluating factor structures, this study seeks to expand evidence for the construct validity of the DERS in forensic samples, thereby contributing to the nomological network of emotion dysregulation in this context. Given the central role of emotion dysregulation as a treatment target and its relevance to risk and recidivism (Seto et al. 2023), clarifying how emotion dysregulation relates to theoretically relevant constructs in forensic populations is of both theoretical and applied importance. At the same time, forensic settings impose unique practical and clinical constraints on assessment. Instruments must not only be psychometrically sound but also clinically feasible for individuals characterized by severe mental illness, cognitive difficulties, and low motivation for assessment. From this perspective, shorter versions of the DERS may offer important advantages. The present study therefore examined the psychometric performance of three versions of the DERS (36‐, 16‐, and 8‐item forms). As such, we aimed at investigating models that may be of particular clinical usefulness.

In the forensic psychiatric sample, externalizing psychopathology and aggressive antisocial behavior were selected to evaluate criterion‐related validity. In the prison sample, impulsivity, aggression, alexithymia, mindfulness, and an alternative measure of ER (the Emotion Regulation Questionnaire, ERQ; Gross and John 2003) were examined to assess criterion‐related and convergent validity. Consistent with theory, we expected differential patterns of association across DERS subscales, with Strategies showing stronger associations with ERQ, Awareness with alexithymia, Impulse with impulsivity, and Nonacceptance with mindfulness. Evidence of construct validity in the forensic psychiatric sample was expected to be reflected in positive associations between DERS scores and externalizing psychopathology and aggressive antisocial behavior. Beyond psychometric adequacy, this approach allows evaluation of whether the DERS functions primarily as a multidimensional profile of emotion dysregulation or, more parsimoniously, as a global measure of such difficulties—an issue with direct implications for theory, intervention planning, and clinical risk assessment.

## 2 Methods

### 2.1 Forensic Psychiatric Sample and Recruitment Procedures

Participants were inpatients consecutively recruited between 2016 and 2020 at a maximum‐security forensic psychiatric hospital in Sweden and were enrolled in the study after providing written, informed consent to participate. Formal ethical approval was granted by the Research Ethics Committee of Linköping University (2016/213‐31 and 2017/252‐32). Eligibility criteria for inclusion were that patients (1) were sentenced to forensic psychiatric care under the Swedish Forensic Mental Care Act and (2) had an expected stay at the hospital of at least 8 weeks. Patients were excluded if they lacked sufficient proficiency in the Swedish language to participate, were judged by their treating psychiatrist as unable to provide informed consent or had an insufficient length of stay. During the recruitment period, 277 patients were available for inclusion. Exclusion criteria reduced the sample size to 184 eligible patients out of which 101 provided informed consent to participate. Three patients withdrew their consent before data collection began. Among the remaining 98 consenting participants, ten patients had missing data across all self‐report measures and were therefore excluded, yielding a final sample of 88 patients. See Figure 1 for a flowchart of the recruitment procedure. The majority of participants were male (*n* = 76, 86%) with a mean age of 35 years (SD = 10.47, range = 20–62) and with a primary diagnosis of schizophrenia spectrum disorder or other psychotic disorders (*n* = 45, 51%). Regarding criminal background, most participants had been convicted of multiple offenses prior to hospitalization (median = 4, range = 1–50), including lethal violence (*n* = 24, 27%), assault (*n* = 73, 83%), sexual offenses (*n* = 12, 14%), theft (*n* = 80, 91%), and drug offenses (*n* = 71, 81%).

**Figure 1:**
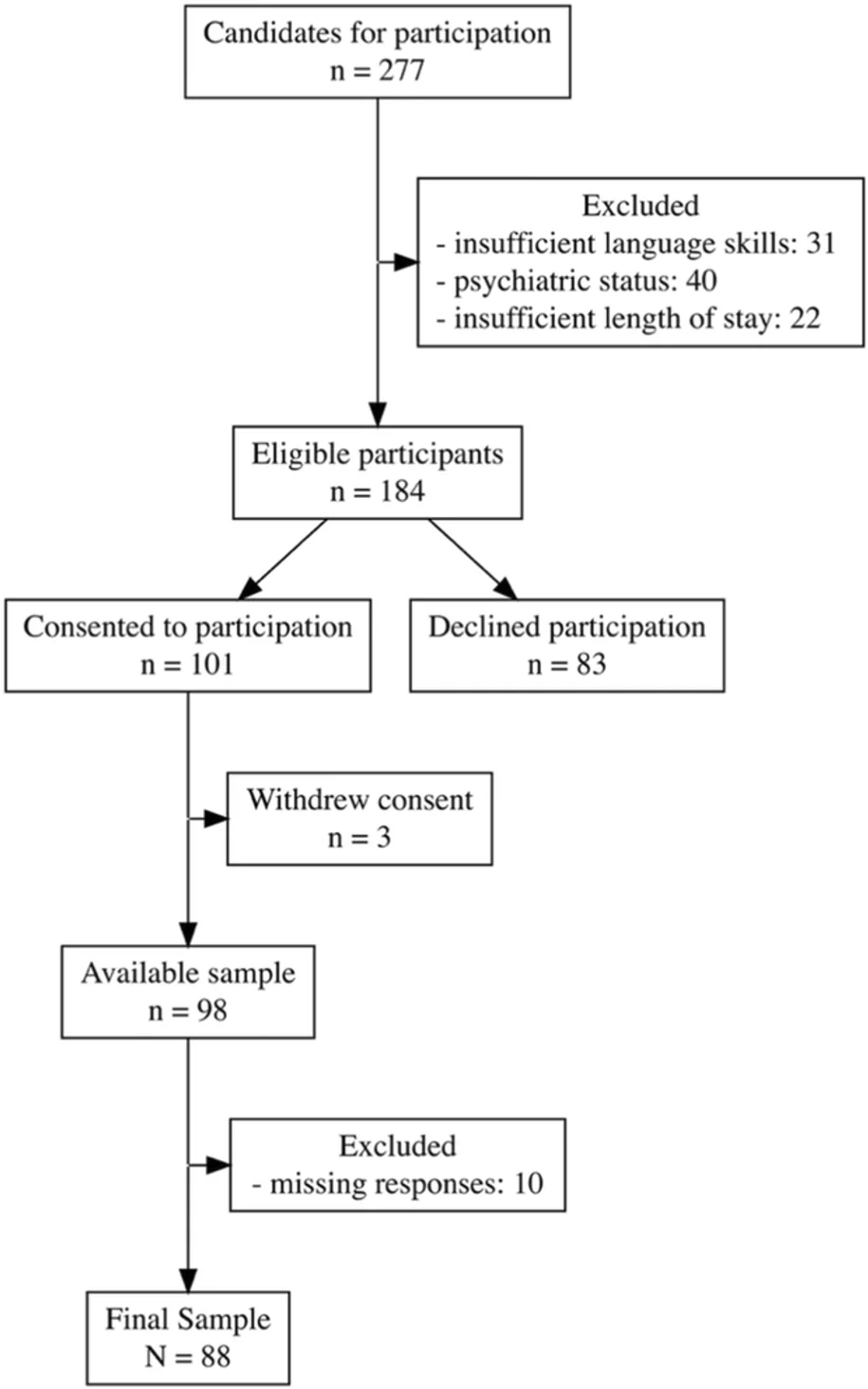
Flowchart of the recruitment procedure for the forensic psychiatric sample.

### 2.2 Prison Sample and Recruitment Procedures

Recruitment of the prison sample was formally approved by the ethics committee of the local university and received authorization from the Italian Ministry of Justice. The sample consisted of individuals incarcerated in 15 medium‐ to large‐sized prisons across Northern and Central Italy. Participants were randomly selected from prison records between March 2013 and July 2015. To be eligible, individuals were required to be fluent in Italian and must not have been taking psychotropic medication during the preceding 3 months. After providing written, informed consent, participants completed assessments in designated rooms typically used for meetings with prison educators. Assessments were conducted individually or in small groups, with group sessions preferred whenever feasible to reduce demands on prison staff. A researcher was present at all times to ensure that participants completed the questionnaires independently. When additional time was required, participants completed assessments across multiple sessions. A total of 389 male offenders participated in the study with a mean age of 40 years (SD = 12.05, range = 19–77). Index crimes included sexual violence or abuse (*n* = 61, 16%), drug‐related violent offenses (*n* = 61, 16%), armed robbery (*n* = 97, 25%), homicide (*n* = 86, 22%), and a mix of minor violent offenses (*n* = 28, 7%). Information on index crime was not available for 56 (14%) participants.

### 2.3 Measures

#### 2.3.1 Difficulties in Emotion Regulation Scale (DERS)

The DERS was administered in both samples in the original 36‐item version (Gratz and Roemer 2004). The DERS provides six subscales with five to eight items in each subscale. These six subscales include Awareness (Item 6; “I am attentive to my feelings”), Clarity (Item 4; “I have no idea how I am feeling”), Impulse (Item 19; “When I'm upset, I feel out of control”), Goals (Item 13; “When I'm upset, I have difficulty getting work done”), Nonacceptance (Item 23; “When I'm upset, I feel like I am weak”), and Strategies (Item 28; “When I'm upset, I believe that there is nothing I can do to make myself feel better”). Items are rated on an ordinal scale from 1 (*almost never*) to 5 (*almost always*). Based on responses to the full DERS‐36, we computed scores for DERS‐16 and DERS‐8. Importantly, DERS‐16 does not retain any items from the Awareness subscale, whereas the remaining subscales are assessed through two to five items. The DERS‐36 was administered in both samples using the Swedish translation in the forensic psychiatric sample (Friberg 2006) and the Italian translation in the prison sample (Giromini et al. 2012). A total of 383 individuals had valid data on DERS items in the prison sample. The corresponding number of valid cases in the forensic psychiatric sample was 88.

#### 2.3.2 Externalizing Spectrum Inventory – Brief Form (ESI‐BF)

The ESI‐BF is a 160‐item self‐report questionnaire aimed at assessing externalizing psychopathology (Patrick et al. 2013). Items are rated from 0 (*not true at all*) to 3 (*completely true*) and summed to provide a total score on 23 facets denoting various forms of externalizing behaviors as well as three broader subfactors labeled General Disinhibition (ESI‐BF_DIS_), Callous Aggression (ESI‐BF_AGG_), and Substance Abuse (ESI‐BF_SUB_). ESI‐BF_DIS_ reflects a broad disinhibitory propensity common across all items of the ESI‐BF whereas the two specific factors, ESI‐BF_AGG_ and ESI‐BF_SUB_, are specific to some items of the questionnaire. ESI‐BF_AGG_ reflects aggressive acts and a lack of remorse and empathy. ESI‐BF_SUB_ assesses recreational and problematic use of marijuana, other drugs and alcohol. The ESI‐BF has previously been used in forensic psychiatric samples (Soe‐Agnie et al. 2016) and prison settings (Driessen et al. 2018). There is sound evidence supporting the reliability and validity of the ESI‐BF in forensic psychiatric settings (Soe‐Agnie et al. 2016; Berlin et al. 2023). The ESI‐BF was administered the forensic psychiatric sample and we relied on factor scores in the analysis as these measure distinct aspects of externalizing psychopathology. Internal consistency for all subfactors exceeded *α* > 0.89 with a total of 87 valid cases.

#### 2.3.3 Life History of Aggression (LHA)

The LHA is an 11‐item, clinician‐rated measure of the lifetime occurrence of aggressive and antisocial behavior (Brown et al. 1982). Items are rated on an ordinal scale ranging from 0 (*no occurrence of the behavior surveyed*) to 5 (*innumerable occurrences of the surveyed behavior*). The questionnaire was rated by one of the two data collectors of the forensic psychiatric sample using available documentation and semi‐structured interviews as data sources. Three subscales can be calculated in the LHA: Aggressive Behavior (LHA_AGG_), Antisocial Behavior (LHA_ANT_), and Self‐directed Aggression (LHA_SH_). LHA has previously been used in Swedish psychiatric and forensic settings (Hofvander et al. 2011; Wallinius et al. 2016) and has evidenced good interrater reliability as well as good convergent validity (Coccaro et al. 1997; Stevanovic et al. 2022). The LHA was administered to the forensic psychiatric sample and both total and subscale scores were calculated and examined individually for a more nuanced analysis. Internal consistency exceeded *α* > 0.78 for all subscales and the composite score with 88 valid cases.

#### 2.3.4 Aggression Questionnaire (AQ)

The AQ is a 29‐item self‐report questionnaire aimed at assessing aggression (Buss and Perry 1992). Participants are asked to rate to what extent each statement applies to them on an ordinal scale ranging from 1 (*extremely uncharacteristic of me*) to 5 (*extremely characteristic of me*). Scores are summed to yield a composite score of overall aggression as well as four subscale scores: Physical aggression, Verbal aggression, Anger, and Hostility. The AQ has been widely applied in forensic psychiatric research (e.g., Garofalo et al. 2022) and the questionnaire's psychometric properties have been extensively investigated across various populations demonstrating good indices of validity and reliability (Diamond et al. 2005; Valdivia‐Peralta et al. 2014). The Italian version, administered to the prison sample, has evidenced good psychometric properties in previous research (Fossati et al. 2003). For simplicity, we relied on the composite score as a measure of trait aggression, with 373 valid cases and internal consistency estimated at *α* = 0.82 in the prison sample.

#### 2.3.5 Toronto Alexithymia Scale (TAS‐20)

The TAS‐20 is a 20‐item self‐report questionnaire designed to measure difficulties in identifying and describing emotions (Bagby et al. 1994). Items are rated on an ordinal scale ranging from 1 (*strongly disagree*) to 5 (*strongly agree*). A total score can be computed as an overall measure, along with three subscales: Difficulties in identifying feelings, Difficulties in describing feelings, and External‐oriented thinking. TAS‐20 has previously been used in prison settings (Roberton et al. 2014; Velotti et al. 2020) with evidence supporting adequate reliability and validity (Preece et al. 2021). The Italian version of TAS‐20 (Bressi et al. 1996), which was used in the present study, has demonstrated reliability and validity consistent with the original measure. However, concerns have been raised regarding the External‐oriented thinking subscale (Kooiman et al. 2002; Preece et al. 2021). For consistency with previous studies employing the TAS‐20 as an external correlate of emotion regulation (e.g., McVey et al. 2022), all subscales were retained, although analyses in the present study focused on the composite score. Internal consistency was estimated at *α* = 0.77, based on 383 valid cases.

#### 2.3.6 Barratt Impulsiveness Scale Version 11 (BIS‐11)

The BIS‐11 is one of the most commonly used measures of impulsivity in psychiatric research (Vasconcelos et al. 2012; Patton et al. 1995). The scale consists of 30 items rated on an ordinal scale ranging from 1 (*rarely*/*never*) to 4 (*almost always*). Three subscales are provided in BIS‐11: Attentional deficits, Motor deficits, and Planning deficits. Items can be summed to yield an overall measure of impulsivity with larger scores indicating greater trait impulsivity. BIS‐11 has demonstrated adequate reliability and validity in previous research (Vasconcelos et al. 2012) which was replicated in the Italian adaptation of the questionnaire that was used in the prison sample (Fossati et al. 2001). For simplicity and in the absence of specific hypothesis at the subscale level, we relied on the composite score as a broad measure of trait impulsivity with 379 valid cases and internal consistency estimated at *α* = 0.80.

#### 2.3.7 Five Facet Mindfulness Questionnaire (FFMQ)

The FFMQ consists of 39 items designed to assess facets of mindfulness (Baer et al. 2006). Items are rated on an ordinal scale ranging from 1 (*never or very rarely true*) to 5 (*very often or always true*) with higher scores indicating greater levels of mindfulness. Five subdomains of mindfulness are quantified in FFMQ: the ability to observe the internal and external world, the ability to describe and label experiences, the ability to act with awareness, a nonjudging stance toward inner experiences, and a nonreacting stance toward negative experiences. The FFMQ has previously been used in forensic settings (Brzozowski et al. 2021) and has demonstrated acceptable psychometric properties (Asensio‐Martínez et al. 2019). The Italian adaptation of the FFMQ, administered in the prison sample, has demonstrated good psychometric qualities in previous studies (Giovannini et al. 2014). In the absence of specific hypothesis at the subscale level and for greater simplicity when interpreting the results, analyses were carried out using the composite score as an overall measure of mindfulness. A total of 360 individuals had valid FFMQ data, and internal consistency was estimated at *α* = 0.77.

#### 2.3.8 Emotion Regulation Questionnaire (ERQ)

The ERQ is a 10‐item self‐report questionnaire assessing the use of two separate ER strategies, namely Cognitive Reappraisal and Expressive Suppression (Gross and John 2003). The ERQ evidenced good psychometric properties in its original version (Gross and John 2003) as well as in the Italian adaptation of the measure (Balzarotti et al. 2010), which was administered to the prison sample in the current study. Items are rated on an ordinal scale ranging from 1 (*strongly disagree*) to 7 (*strongly agree*) with six items comprising the Cognitive Reappraisal subscale and four items the Expressive Suppression subscale. Summing items to a composite score is not advisable as the strategies measured in the ERQ are theoretically positioned to measure adaptive and maladaptive ER strategy use (Gross and John 2003). Guided by previous research we had specific hypotheses at the subscale level why analyses were carried out for Expressive Suppression and Cognitive Reappraisal individually. Internal consistency was estimated at *α* = 0.68 for Expressive Suppression and *α* = 0.82 for Cognitive reappraisal with a total of 362 valid cases for both subscales.

### 2.4 Missing Data

#### 2.4.1 Forensic Psychiatric Sample

The average rate of missing data in the forensic psychiatric sample (*n* = 88) was below 1% and 78 cases (89%) were complete across all variables with 87% of cases being complete for the subset of the data containing DERS items. The few missing data points within this data set were imputed through the expectation‐maximization algorithm for each questionnaire independently.

#### 2.4.2 Prison Sample

In the Italian prison sample (*n* = 389), the average rate of missingness across the entire data set was 3.6% with a range of missingness across items of 0.0%–8.2%. A total of 191 (49%) cases had complete data across all variables. Importantly, 83% of cases were complete for the subset of the data containing the DERS items. Cases that had missing values equal to or exceeding 20% on each questionnaire were removed. In cases with < 20% missing data, the expectation‐maximization algorithm was used to impute missing values.

### 2.5 Statistical Analysis

Data from two independent samples were used in this study and statistical procedures were adapted to each sample's characteristics and attributes. In both samples, the same procedures were applied to handle missing data (e.g., the expectation‐maximization algorithm). However, due to sample size differences, different estimators are used in the CFA for the two samples. Likewise, different criterion variables are used to assess validity. See subheadings below for more details on statistical procedures for each sample. All code used in the analyses is freely and publicly available on the Open Science Framework (OSF): https://osf.io/xd2fc/overview?view_only=768ea14288c84609bfa31c38bc1787da.

#### 2.5.1 Confirmatory Factor Analyses

CFA were performed in the R programming language (R Core Team 2023) using the Lavaan package (Version 0.6‐17; Rosseel 2012). We aimed to replicate structural models previously reported in the literature for the full‐length DERS and two short forms, DERS‐16 and DERS‐8. The original six‐factor correlated traits model was fitted for DERS‐36 which has been replicated in previous studies (e.g., McVey et al. 2022; Neumann et al. 2010; Perez et al. 2012). A five‐factor structure was fitted for DERS‐16 which has been supported in previous studies (Lawlor et al. 2021). Finally, a unidimensional model was investigated for the more recently developed DERS‐8 (Penner et al. 2023). We opted for weighted least squares (WLS) estimation, as this approach accounts for differences in residual variances when estimating model parameters and is recommended for ordinal data (Rhemtulla et al. 2012). However, due to the large sample size requirements associated with WLS estimation, it was not possible to estimate the six‐factor correlated traits model and the five‐factor model in the forensic psychiatric sample. Consequently, robust maximum likelihood estimation (MLR) was used for the forensic psychiatric sample, as MLR is more efficient and robust in smaller samples. All models were constrained with factor variances set to one allowing factor loadings to be freely estimated. Model fit was evaluated using multiple fit indices, including the robust comparative fit index (CFIr), the robust Tucker–Lewis index (TLIr), the root mean square error of approximation (RMSEA), and the standardized root mean square residual (SRMR). In line with commonly cited guidelines, RMSEA values below 0.08 and 0.06 were interpreted as indicating adequate and good model fit, respectively (J. Wang and X. Wang 2012). CFIr and TLIr values above 0.90 and 0.95 were used as benchmarks for adequate and good model fit, respectively, and SRMR values below 0.08 were considered indicative of good model fit (Hu and Bentler 1999).

#### 2.5.2 Reliability Analysis

Reliability for subscales and total scores was primarily estimated through McDonald's omega (*ω*; McDonald 1999). Omega can be conceived of as a weighted alpha coefficient that takes into account several sources of variance in the estimation of reliability. Similar to Cronbach's alpha (*α*; Cronbach 1951), *ω* ranges from 0 (*no reliability*) to 1 (*perfect reliability*) and is a model‐based reliability estimate. We preferred estimating reliability through *ω* as the assumptions underlying *α* tend to bias the estimate (McNeish 2018). Previous factor‐analytic studies on DERS evidence that tau‐equivalence cannot be assumed (e.g., McVey et al. 2022) as the factor loadings vary across items which favors the use of *ω* over *α*. Another advantage of *ω* is the possibility to model different sources of variance and to estimate reliability taking different sources of variance into account (Flora 2020). These properties of *ω* make it preferable over *α* as a measure of internal consistency when measures are multidimensional (McNeish 2018; Flora 2020). However, for the sake of continuity with previous research, estimates of *α* are reported alongside *ω* values.

#### 2.5.3 Validity Analysis

We aimed to examine construct validity, looking at both convergent validity and divergent validity, for those models that showed at least acceptable structural fit. In the forensic psychiatric sample, LHA and ESI‐BF were used as criterion variables. In the Italian prison sample, validity was assessed through concurrent associations with ERQ, TAS‐20, BIS‐11, FFMQ, and AQ. We propose that effect sizes in the correlational analysis are to be informed by what is typically found within psychological research with *r* = 0.1 being a small effect size, *r* = 0.2 being a medium effect size, and *r* = 0.3 being a large effect size (Funder and Ozer 2019). Guided by previous literature, effect sizes in this study will be discussed using previous research findings to contextualize the magnitude and importance of the effect sizes. Due to the skewed distributions of the DERS scores across samples (see Figure S1 for distributions within each sample), Spearman's *ρ* was used in all correlation analyses.

## 3 Results

### 3.1 Descriptive Statistics and Reliability Estimates

Descriptive statistics and reliability estimates are provided in Table 1 for both samples across all models. Across both samples, *α* and *ω* values yielded similar reliability estimates for DERS‐36. However, estimates were markedly lower for the Awareness subscale. Reliability estimates for DERS‐16 were slightly lower, but all *α* values exceeded 0.71, and all *ω* values exceeded 0.72 in the forensic psychiatric sample. DERS‐8 was found to have good internal consistency as indicated by both *α* and *ω* values. Average inter‐item correlations ranged from 0.22 to 0.60 for DERS‐36 and from 0.39 to 0.67 for DERS‐16, and were estimated at 0.44 for DERS‐8 in the forensic psychiatric sample. Similar descriptive statistics and reliability estimates were found in the prison sample (see Table 1). Measures of central tendency were lower in the prison sample, and lower average inter‐item correlations were observed across all DERS forms. The dispersion of the data was greater in the forensic psychiatric sample as indicated by larger interquartile ranges and standard deviations compared to the prison sample. The DERS exhibited similar distributions across the three forms and a graphical depiction of the distribution of DERS‐36 for both samples is provided in the Supporting Material (see Figure S1).

**Table 1:** Descriptive statistics and reliability estimates across samples and different forms of the Difficulties in Emotion Regulation Scale.

	*N*_items_	*M*	Md	SD	IQR	*M*_R_	*α*	*ω*
Forensic psychiatric sample (*N* = 88)								
DERS‐36	36	81.94	76.57^a^	25.53	40.5	0.28	0.94	0.95
Strategies	8	18.02	16.5^a^	6.88	9.25	0.34	0.80	0.81
Nonacceptance	6	12.39	10^a^	6.28	10	0.57	0.89	0.89
Impulse	6	13.18	12^a^	6.31	11.25	0.6	0.90	0.91
Goals	5	15.45	16^a^	5.76	10	0.59	0.88	0.88
Awareness	6	13.97	14	4.51	7	0.22	0.63	0.54
Clarity	5	8.93	8	3.81	5	0.42	0.79	0.79
DERS‐16	16	36.88	34^a^	14.36	20	0.42	0.92	0.94
Strategies	5	10.95	10^a^	4.79	7.25	0.39	0.76	0.77
Nonacceptance	3	6.15	5	3.3	5.14	0.57	0.80	0.82
Impulse	3	6.86	6^a^	3.6	5.25	0.67	0.86	0.87
Goals	3	9.24	9^a^	3.67	7	0.61	0.82	0.83
Clarity	2	3.67	3	1.89	3	0.56	0.71	0.72
DERS‐8	8	19.02	17.5^a^	7.54	11	0.44	0.86	0.87
Prison sample (*N* = 383)								
DERS‐36	36	74.44	71^a^	19.86	27	0.22	0.90	0.97
Strategies	8	14.78	13^a^	5.93	8	0.39	0.83	0.86
Nonacceptance	6	13.57	13^a^	5.5	7	0.43	0.82	0.84
Impulse	6	11.08	10^a^	4.58	5	0.43	0.80	0.85
Goals	5	11.76	11^a^	4.35	5	0.38	0.75	0.79
Awareness	6	14.21	14	4.16	6	0.22	0.61	0.07
Clarity	5	9.04	8	3.57	5	0.35	0.73	0.76
DERS‐16	16	31.08	28^a^	12.11	15	0.41	0.92	0.95
Strategies	5	9.05	8^a^	4.18	5	0.47	0.82	0.83
Nonacceptance	3	6.28	6	2.99	4	0.49	0.74	0.74
Impulse	3	5.1	4^a^	2.75	3	0.64	0.84	0.87
Goals	3	6.83	6^a^	2.97	3.5	0.51	0.75	0.76
Clarity	2	3.82	3	1.94	3	0.53	0.69	0.70
DERS‐8	8	15.86	14^a^	6.36	9	0.41	0.85	0.87

Abbreviations: *α*, Cronbach's alpha; DERS, Difficulties in Emotion Regulation Scale; IQR, interquartile range; *M*_R_, average inter‐item correlations; *ω*, McDonald's omega.

^a^ Indicates that the two samples differ in the nonparametric Wilcoxon rank‐sum test on the respective DERS scale.

### 3.2 Factor Structure of DERS‐36

The six‐factor correlated traits model showed poor fit across all fit indices in both samples as displayed in Table 2. Factor loadings in the forensic psychiatric sample averaged 0.66 (range: 0.03–0.87) and 0.65 (range: ‐0.50–0.89) in the prison sample. Lower factor loadings were estimated for those items modeled on awareness in both samples (range in forensic psychiatric sample: 0.03–0.68; range in prison sample: 0.03–0.68). Notably, Item 17 was not a significant indicator of awareness in either sample. Moreover, Item 34 was estimated with a negative factor loading when modeled on awareness in the prison sample. The average inter‐factor correlation in the forensic psychiatric sample was estimated at *r*_*Mean*_ = 0.55 and at *r*_*Mean*_ = 0.63 in the prison sample. Notably, the awareness factor had low to nonsignificant relationships with the remaining factors as indicated by the covariance matrix for the latent variables. Modification indices in the forensic psychiatric sample suggested residual covariances between several item pairs (Item 36 ~ Item 12, Item 12 ~ Item 10, Item 27 ~ Item 2). Modification indices for the prison sample suggested that cross‐loadings over all latent variables existed for Items 34, 17, and 7.

**Table 2:** Fit indices for all confirmatory factor analysis models.

Model	*k*	df	CFIr	TLIr	RMSEA [95% CI]	SRMR
Forensic psychiatric sample (*N* = 88)						
DERS‐36	87.00	579.00	0.76	0.74	0.09 [0.08, 0.1]	0.09
DERS‐16	42.00	94.00	0.99	0.98	0.03 [0, 0.07]	0.06
DERS‐8	16.00	20.00	0.93	0.90	0.1 [0.05, 0.15]	0.06
Prison sample (*N* = 383)						
DERS‐36	195.00	579.00	0.70	0.67	0.11 [0.11, 0.12]	0.10
DERS‐16	90.00	94.00	0.87	0.83	0.13 [0.11, 0.14]	0.05
DERS‐8	40.00	20.00	0.92	0.88	0.13 [0.1, 0.15]	0.05

*Note:* Number of free parameters (*k*) differ between samples due to different estimation methods.

Abbreviations: CFIr, Comparative Fit index (robust); df, degrees of freedom; *k*, number of free parameters; RMSEA, root mean square error of approximation; SRMR, standardized root mean square residual; TLIr, Tucker–‐Lewis index (robust).

### 3.3 Factor Structure of DERS‐16

Fit indices were markedly improved for DERS‐16 in both samples. Fit measures indicated good model fit in the forensic psychiatric sample. However, only the SRMR showed acceptable fit in the prison sample. Although remaining fit measures did not reach thresholds of acceptable fit, the model was markedly improved through CFI and TLI values. For consistency with the forensic psychiatric sample, factor loadings for DERS‐16 are displayed in Table 3 for both samples. The average factor loading in the forensic psychiatric sample was 0.73 (range: 0.56–0.88) and 0.79 (range: 0.68–0.91) in the prison sample. Inter‐factor correlations were estimated at *r*_*Mean*_ = 0.68 for the forensic psychiatric sample and *r*_*Mean*_ = 0.75 for the prison sample. Modification indices for the prison sample suggested that Item 16 had cross‐loadings on impulse and strategies and a correlated residual with Item 15.

**Table 3:** Factor loadings for DERS‐16 in both samples.

Five‐factor model						
Item_no_	*λ*_Strategies_	*λ*_Nonacceptance_	*λ*_Impulse_	*λ*_Goals_	*λ*_Clarity_	Θ
Forensic psychiatric sample (*N* = 88)						
15	0.56 (0.11)					0.68 (0.12)
16	0.61 (0.08)					0.62 (0.1)
28	0.64 (0.08)					0.59 (0.1)
30	0.58 (0.09)					0.67 (0.1)
36	0.75 (0.07)					0.44 (0.1)
21		0.69 (0.09)				0.53 (0.12)
23		0.78 (0.08)				0.39 (0.12)
29		0.82 (0.06)				0.33 (0.1)
14			0.84 (0.07)			0.3 (0.11)
19			0.87 (0.05)			0.24 (0.08)
27			0.76 (0.08)			0.42 (0.11)
13				0.67 (0.08)		0.55 (0.11)
18				0.81 (0.05)		0.35 (0.08)
33				0.88 (0.04)		0.23 (0.07)
5					0.66 (0.15)	0.56 (0.19)
9					0.84 (0.13)	0.29 (0.21)
Prison sample (*N* = 383)						
15	0.75 (0.03)					0.43 (0.05)
16	0.74 (0.03)					0.45 (0.04)
28	0.69 (0.03)					0.52 (0.05)
30	0.86 (0.02)					0.26 (0.04)
36	0.79 (0.03)					0.38 (0.04)
21		0.71 (0.03)				0.5 (0.05)
23		0.72 (0.03)				0.48 (0.05)
29		0.84 (0.03)				0.29 (0.04)
14			0.89 (0.02)			0.21 (0.04)
19			0.91 (0.02)			0.17 (0.04)
27			0.83 (0.02)			0.31 (0.04)
13				0.68 (0.03)		0.54 (0.05)
18				0.77 (0.03)		0.41 (0.04)
33				0.86 (0.03)		0.26 (0.04)
5					0.72 (0.04)	0.48 (0.05)
9					0.85 (0.03)	0.29 (0.06)

Abbreviations: *λ*_Clarity_, factor loadings on subscale Clarity; *λ*_Goals_, factor loadings on subscale Goals; *λ*_Impulse_, factor loadings on subscale Impulse; *λ*_Nonacceptance_, factor loadings on subscale Nonacceptance; Θ, residual variance; *λ*_Strategies_, factor loadings on subscale Strategies.

### 3.4 Factor Structure of the DERS‐8

Fit indices produced divergent results for DERS‐8 in both samples. Whereas good model fit was found through CFI, TLI, and SRMR values in the forensic psychiatric sample, the RMSEA did not indicate acceptable fit. In the prison sample, acceptable model fit was found according to CFI and SRMR values, whereas TLI and RMSEA values did not reach the threshold for acceptable fit. The average factor loading in the forensic psychiatric sample was 0.66 (range: 0.47–0.74) and 0.71 (range: 0.58–0.82) in the prison sample (see Table 4). Modification indices for the forensic psychiatric sample suggested the presence of correlated residuals for two item pairs (Item 19 ~ Item 27 and Item 21 ~ Item 29). Similarly, modification indices for the prison sample indicated correlated residuals for three item pairs (Item 21 ~ Item 29, Item 19 ~ Item 27, and Item 21 ~ Item 27).

**Table 4:** Factor loadings for DERS‐8 in both samples.

One‐factor model		
Item_no_	*λ*_DERS_	Θ
Forensic psychiatric sample (*N* = 88)		
13	0.64 (0.08)	0.60 (0.10)
19	0.73 (0.08)	0.47 (0.11)
21	0.66 (0.10)	0.57 (0.13)
27	0.69 (0.09)	0.52 (0.13)
28	0.63 (0.08)	0.61 (0.11)
29	0.74 (0.07)	0.46 (0.11)
33	0.74 (0.06)	0.45 (0.09)
35	0.47 (0.11)	0.78 (0.11)
Prison sample (*N* = 383)		
13	0.58 (0.04)	0.66 (0.04)
19	0.82 (0.03)	0.32 (0.04)
21	0.63 (0.04)	0.60 (0.05)
27	0.80 (0.02)	0.36 (0.04)
28	0.65 (0.04)	0.58 (0.05)
29	0.79 (0.03)	0.38 (0.04)
33	0.76 (0.03)	0.42 (0.04)
35	0.66 (0.03)	0.56 (0.05)

Abbreviations: *λ*_DERS_, factor loadings on the latent variable; Θ, residual variance.

### 3.5 Validity

We proceeded to investigate validity for those models that exhibited at least acceptable model fit according to our a priori benchmarks. Although only the SRMR value indicated acceptable model fit for DERS‐16 in the prison sample we proceeded with investigating validity for this model, as considerations of validity should not rest solely on fit measures and a priori thresholds. In sum, we proceeded with investigating validity for DERS‐16 and DERS‐8 in both samples while disregarding DERS‐36, as the six‐factor model showed poor fit with negative and nonsignificant factor loadings in both samples. A correlation plot for each sample is provided in Figures 2 and 3 for the forensic psychiatric sample and the prison sample, respectively.

**Figure 2:**
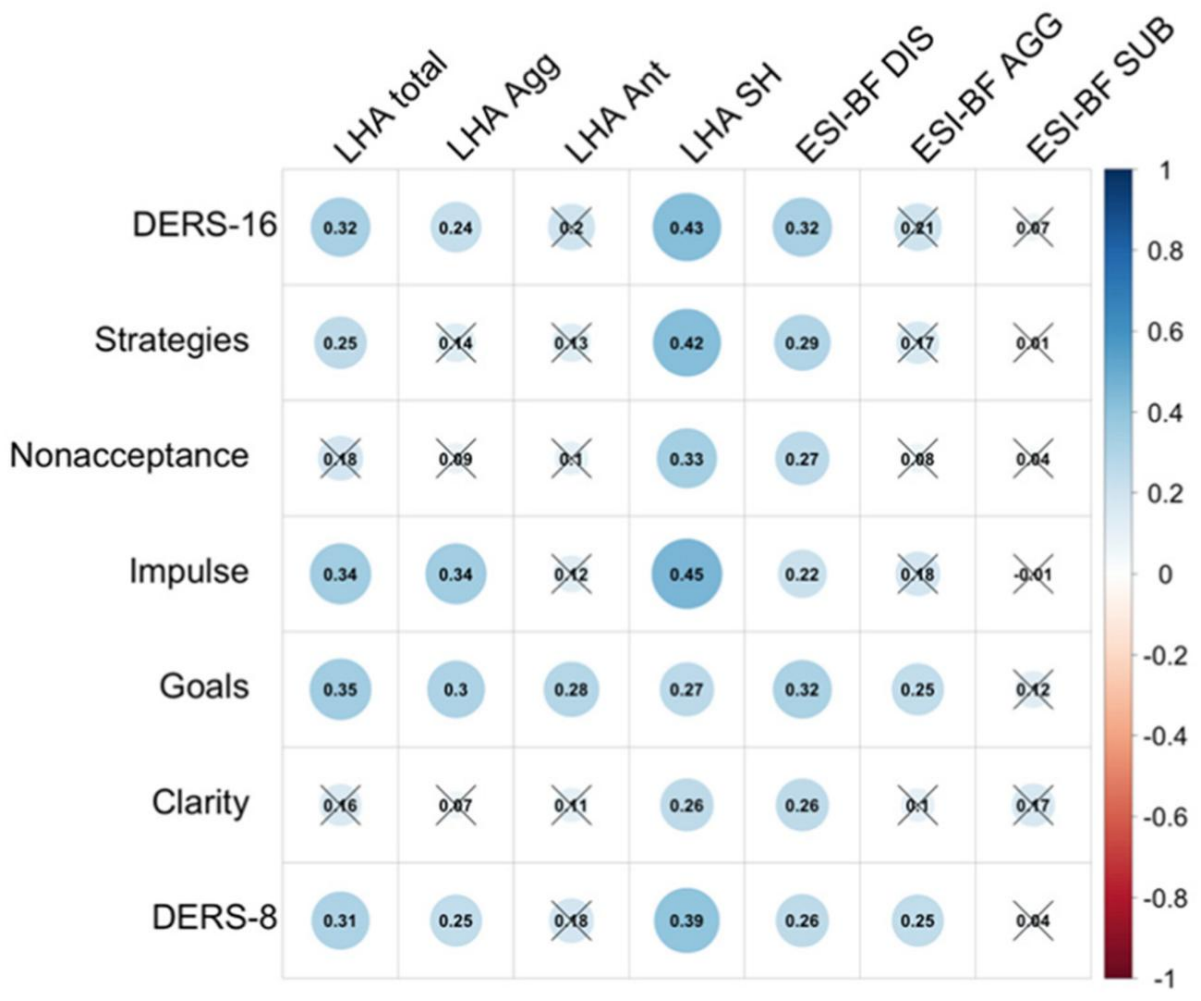
Correlation plot for forensic psychiatric sample. LHA, Life History of Aggression; ESI‐BF, Externalizing Spectrum Inventory‐Brief Form; DERS, Difficulties in Emotion Regulation Scale. Crossed cells are nonsignificant correlations with *p* > 0.05.

**Figure 3:**
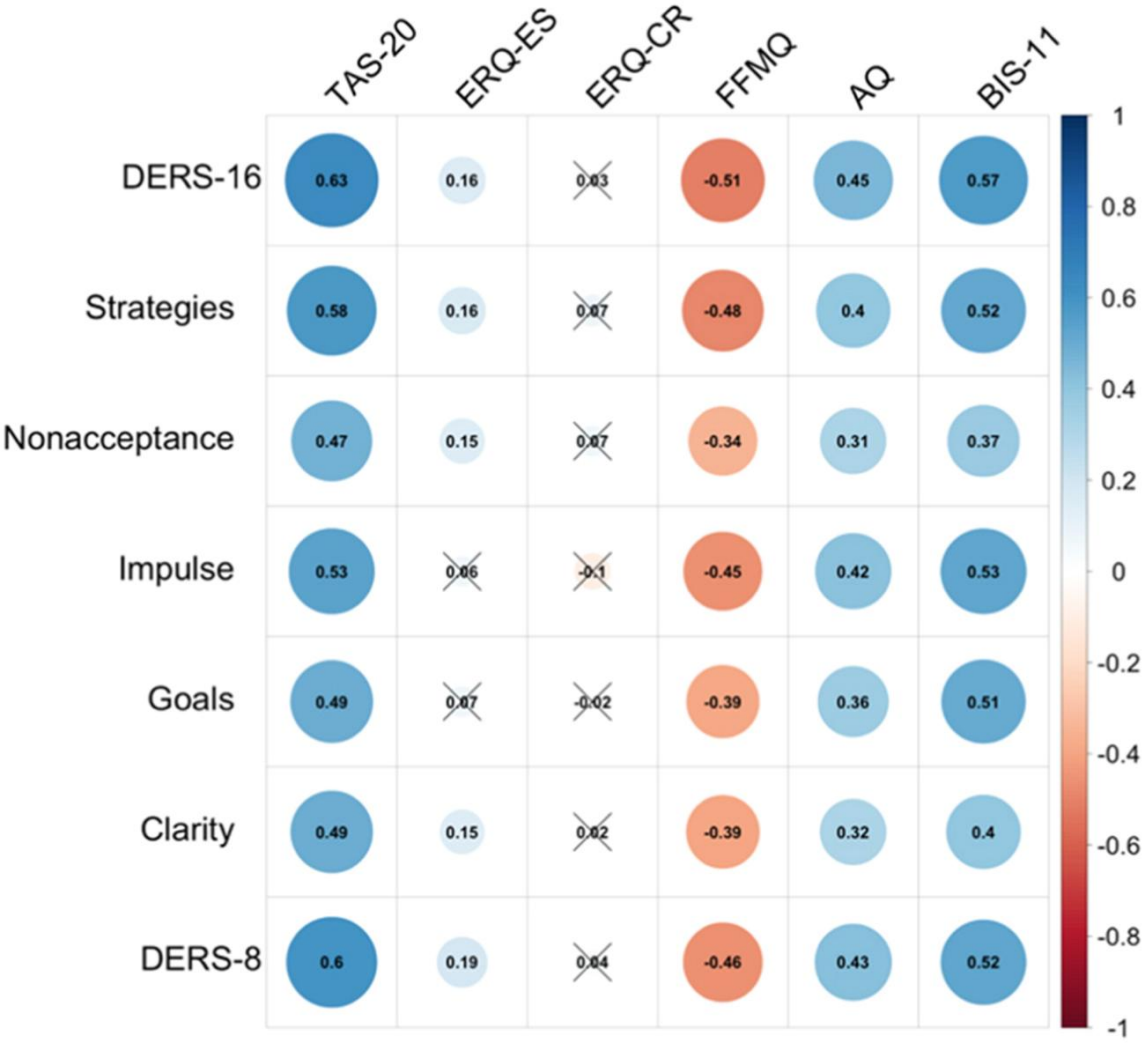
Correlation plot for prison sample. DERS, Difficulties in Emotion Regulation; TAS‐20, Toronto Alexithymia Scale; ERQ‐ES, Suppression; ERQ‐CR, Cognitive Reappraisal; FFMQ, Five Facet Mindfulness Questionnaire; AQ, Aggression Questionnaire; BIS‐11, Barratt Impulsiveness Scale. Crossed cells are nonsignificant correlations with *p* > 0.05.

The pattern of correlations for DERS‐16 in the forensic psychiatric sample showed significant associations with most external correlates except for Antisocial Behavior, Callous Aggression, and Substance Abuse. The Nonacceptance and Clarity subscales were only associated with Self‐harm and General Disinhibition, whereas the Goals subscale was most consistently associated with all external correlates. In the prison sample, a rather consistent pattern of associations was found between DERS‐16 and external correlates. Notably, no significant association was found between DERS‐16 and Cognitive Reappraisal. With two exceptions, the remaining associations were in the expected directions and significant.

DERS‐8 was related to all external correlates in the forensic psychiatric sample except Antisocial Behavior and Substance Abuse and was similarly associated with all external correlates in the prison sample except for Cognitive Reappraisal.

## 4 Discussion

DERS is a widely used self‐report measure for assessing emotion dysregulation. While several psychometric evaluations have been conducted over the years—considering various models in both clinical and nonclinical samples—no study has systematically evaluated the DERS in a forensic psychiatric or prison sample, where reliability, validity, and factor structure are examined together. This represents an important knowledge gap considering the increasing use of the DERS and its derivatives in forensic settings and its potential relevance for clinical practice. The present study is the first, to our knowledge, that evaluated the reliability, validity, and structure of the original DERS‐36 and two short forms (DERS‐16 and DERS‐8) within a single study, using two independent forensic samples drawn from two different settings across two different countries. Beyond psychometric adequacy, the present study offers new insights into the clinical utility of the DERS and its short forms in forensic settings, with implications for assessment feasibility and practical application.

### 4.1 Structural Fit and Reliability

Analysis revealed poor fit for the six‐factor correlated traits model across both samples. However, reliability estimates for the composite score indicated good internal consistency and precision. Reliability estimates were also high for individual subscales, although lower for Awareness in both samples (*α* < 0.70, *ω* < 0.55). The markedly low *ω* value in the prison sample is likely due to negative factor loadings for two items (Item 34 and Item 17) in the Awareness subscale. Interestingly, Item 17 (“when I'm upset, I believe that my feelings are valid and important”) was not a significant indicator of Awareness in either sample. Importantly, our results suggest that Awareness may not share the same sound psychometric qualities as the remaining subscales of the DERS.

Consistent with previous research investigating the psychometric properties of a six‐factor solution, low convergence was found between Awareness and remaining subscales in our two samples (Hallion et al. 2018; Osborne et al. 2017). One possible interpretation of this is that the Awareness subscale diverges conceptually from the remaining DERS subscales that tend to cluster as evidenced by high inter‐factor correlations. As opposed to the other DERS subscales that assess how individuals *react* to their emotions, Awareness assesses the extent to which individuals *notice* their emotions which has been put forth as an explanation for the low convergence of the Awareness subscale (Hallion et al. 2018). However, similar to the items in the Awareness subscale, the Clarity subscale does not assess how individuals react to emotions, but rather the granularity of their emotional perception and as such is related to Awareness. Yet, Clarity does not exhibit the same form of divergence from other DERS subscales. An alternative explanation for the lower convergence for the Awareness subscale may be found in methodological artefacts, rather than in conceptual divides. As noted by Bardeen et al. (2012), all items in the Awareness subscale are reverse‐coded which may contribute to inflated error and low convergence with other subscales of the measure. In a follow‐up study, Bardeen et al. (2016) found that Clarity and Awareness items loaded on one common factor that they labeled “Identification.” This is similar to how emotion dysregulation was conceptualized in the original article by Gratz and Roemer (2004) prior to conducting the factor analysis. Interestingly, we found, for both samples, that the Awareness subscale was most strongly correlated with the Clarity subscale. Albeit hypothetically, it is possible that Awareness may be conceptually distinct from the remaining DERS subscales and that Clarity, to some extent, may act as a bridge between Awareness and the remaining DERS subscales.

The DERS‐16 showed good model fit in the forensic psychiatric sample and fit indices improved in the prison sample although they did not reach a priori thresholds. All factor loadings exceeded 0.5 with 32%–77% of item variance being attributable to the latent variables. Reliability estimates, both *α* and *ω* values, indicated good reliability for both the composite score and the subscale scores and are in line with findings from previous studies that have reported good psychometric qualities for DERS‐16 in similar clinical samples (e.g., Lawlor et al. 2021). There is currently no consensus on whether the composite score or subscale scores are to be used for DERS. To gauge the advantage of using the composite or subscale scores, we investigated the validity of both, which is further discussed in the paragraph below. However, from a statistical point of view, relying on subscale scores for DERS‐16 is supported by the data as evidenced by the fit indices and estimates of reliability. Arguably though, the decision of whether to use the composite score or subscale scores should be guided as much by theoretical and substantial considerations as by psychometric evidence. The inter‐factor correlations in DERS‐16 suggest that the different subscales are indeed empirically related, as do the reliability estimates for the composite score. This highlights that the composite score is a parsimonious and efficient measure of all those processes implied to constitute ER within the DERS framework.

Moving forward, we found diverging evidence in terms of fit indices for DERS‐8. In the forensic psychiatric sample, all fit indices except RMSEA tended to support the structural integrity of a one‐factor solution. Evidence was harder to evaluate in the prison sample as two fit indices indicated poor fit and two indicated adequate and good fit, respectively. In terms of reliability, estimates for DERS‐8 remained satisfactory. Perhaps contributing to this may be that all DERS‐8 items provide respondents with the same uniform context. All DERS‐8 items start with “When I'm upset…” and as such provide the same context within which respondents are asked to evaluate their ER skills.

Overall, the fit indices yielded divergent results across samples and models. The relatively low CFI and TLI values observed for the DERS‐36 model may be explained by lower average inter‐item correlations, likely reflecting the weak convergence between the Awareness subscale and the remaining DERS dimensions. As average inter‐item correlations increased in the DERS‐16 and DERS‐8 models, CFI and TLI values improved accordingly. RMSEA values, in contrast, remained consistently elevated across models and samples, with the DERS‐16 in the forensic psychiatric sample constituting a notable exception. This pattern can be understood in light of how RMSEA is calculated. RMSEA estimates the amount of systematic model misspecification per degree of freedom (df) in the population, making it particularly sensitive to many small, repeated misspecifications in large and highly constrained models such as the DERS‐36. In addition, larger sample sizes provide greater statistical power to detect even minor deviations between the observed and model‐implied covariance matrix, which is likely to contribute to the elevated RMSEA values observed in the prison sample. Conversely, smaller sample sizes reduce the stability of *χ* ^2^‐based fit indices—such as the RMSEA—which may contribute to greater variability in RMSEA values across models in the forensic psychiatric sample. The comparatively low RMSEA value for the DERS‐16 model in the forensic psychiatric sample is likely attributable to both the removal of the Awareness items—identified as a potential source of misspecification—and the reduced degrees of freedom of the model. The elevated RMSEA value observed for the more parsimonious DERS‐8 may further reflect the rigidity of a unidimensional model, in which strong constraints limit the model's capacity to absorb minor misspecifications. In contrast, SRMR values followed a more consistent pattern across samples, with higher values observed for the DERS‐36 model and lower values for the DERS‐16 and DERS‐8 models. SRMR reflects the average standardized difference between observed and model‐implied correlations (Goretzko et al. 2024) and, unlike RMSEA, does not penalize large or highly constrained models to the same extent. Consequently, SRMR is less sensitive to the accumulation of small, systematic misspecifications that can substantially inflate RMSEA values.

### 4.2 Validity

Despite mixed findings regarding the factor structure of the DERS and its derivatives in our samples, conclusions regarding the quality of psychometric measures should also include considerations of their construct validity and not rely only on factor analytic results (Sellbom and Tellegen 2019). First, we aim to discuss and interpret findings from the forensic psychiatric sample, followed by the findings from the prison sample.

The correlation plot for the forensic psychiatric sample displays a rather consistent pattern, with significant associations observed across all external correlates except for Antisocial behavior, Callous Aggression, and Substance Abuse. DERS‐16 correlated with several criterion variables with medium to large effect sizes, in line with what would be expected by theory and previous research. The effect size between the DERS‐16 composite score and aggression is similar in magnitude to that reported between other DERS short forms and aggression (Facer‐Irwin et al. 2023) as well as that reported between DERS‐36 and aggression (Garofalo et al. 2021) and DERS‐36 and intimate partner violence (Gratz and Roemer 2004). Somewhat surprisingly, DERS‐16 did not covary with the ESI‐BF_SUB_ as would be expected given previous research (e.g., Stellern et al. 2023) and theory (Roos and Kober 2024). One tentative explanation for this may be that the items in the Substance Abuse scale primarily assess behavioral components of substance abuse rather than emotional processes sustaining substance abuse (e.g., craving; Roos and Kober 2024). Also, it is important to consider that the correlations in the forensic psychiatric sample are based on a relatively small sample size and should be interpreted with some caution. The effect size of the relationship between DERS‐16 and the General Disinhibition scale was similar in magnitude to that found in an adolescent psychiatric sample (Penner et al. 2023). Importantly, correlations were generally similar for DERS‐16 and DERS‐8 in the forensic psychiatric sample, indicating that the brevity of DERS‐8 does not compromise its validity.

The correlation plot for the Italian prison sample showed a consistent pattern of associations with larger effect sizes than those observed in the forensic psychiatric sample. The larger effect sizes may partly reflect that several external correlates in the prison sample partly overlap with ER processes. For instance, effective ER hinges on an individual's ability to appraise and understand emotions, an ability also implicated in the construct of alexithymia (Preece et al. 2023). Both DERS‐16 and DERS‐8 were associated with measures of alexithymia, mindfulness, impulsivity, and aggression. Expressive Suppression was, however, not related to Impulse or Goals, and Cognitive Reappraisal was not significantly related to any subscale or composite score of the DERS‐16 and DERS‐8. Theoretically, Cognitive Reappraisal should be negatively related to measures of dysfunctional ER since cognitive reappraisal has been conceptualized as an adaptive ER strategy (Gross and John 2003). The use of cognitive reappraisal and the presence of dysfunctional ER skills may partly reflect different processes and may not necessarily mirror one another. In other words, one could have dysfunctional ER skills (as measured through higher DERS scores) whilst this does not have to entail anything about the frequency of cognitive reappraisal (i.e., scores on the Cognitive Reappraisal subscale). This may explain the lack of association between Cognitive Reappraisal and DERS in the prison sample. Perhaps more important is the extent to which one is effective in regulating emotions rather than whether one uses a purportedly adaptive ER skill or not (Rogier et al. 2019).

A second noteworthy finding from the prison sample is the consistency of correlations across the various subscales. Contrary to what we would expect, we did not find relatively stronger associations between Nonacceptance and mindfulness or between Impulse and impulsivity (as measured by the BIS‐11). As such, evidence of validity, conceptualized as correlations with theoretically adjacent constructs, is equally strong for all subscales in DERS‐16. The lack of differentiation between DERS subscales and our external correlates may suggest that the composite score is a more parsimonious measure than subscale scores.

### 4.3 Sample Characteristics and Clinical Implications

Differences in sample characteristics—beyond sample size—should be considered when interpreting the present findings. The forensic psychiatric sample was characterized by severe mental illness, with more than half of the participants diagnosed with a psychotic disorder. Although impaired insight is a core feature of psychosis, prior research suggests that individuals with psychotic disorders can nonetheless provide reliable and valid self‐reports of emotional and psychological functioning (e.g., Bell et al. 2007). From this perspective, access to psychometrically sound and clinically feasible self‐report instruments is essential to ensure that patients' subjective functioning is adequately represented in both clinical practice and research within forensic psychiatric settings.

Moreover, other differences between our two samples, such as cultural differences and medical regimens, may further complicate direct comparisons of our results and should be taken into account when appraising the results. For instance, emotional numbness is a common side‐effect reported amongst users of antipsychotic medication (Read and Williams 2019), which is prescribed to the large majority (93% in 2024) of forensic psychiatric patients in Sweden (Swedish National Forensic Psychiatric Register 2024). Similarly, cultural differences between Sweden and Italy may contribute to the overall findings in this study, although both countries are Western European. As previous research has highlighted, ER is contextual (Aldao and Tull 2015), which is why comparisons across settings and samples always should be made with caution.

Previous research on people receiving forensic psychiatric care has shown that low levels of literacy skills (Svensson et al. 2015) and low educational attainment (Laporte et al. 2021) are common. Similar findings have been reported in prison populations (Morken et al. 2021). Given this, the use of DERS short forms may be particularly useful in these settings as their—relative—brevity does not tax patients' and participants' reading comprehension to the same extent as full forms, which may enhance the quality of self‐report measures more broadly. Brief measures such as the DERS‐16 and DERS‐8 may therefore be more feasible for routine clinical use, for example, during intake assessments, repeated monitoring of treatment progress, or structured risk formulation. Further adding to the clinical usefulness of DERS short‐forms is the limited differentiation between specific ER processes as measured by the DERS in both samples. This suggests that, in forensic contexts, the DERS may primarily function as a measure of global emotion dysregulation rather than a finely differentiated profile of emotion dysregulation. Under such conditions, the use of composite scores or brief unidimensional forms may be clinically parsimonious.

### 4.4 Strengths and Limitations

Although contributing new knowledge about DERS within forensic settings, the findings should be interpreted with consideration to the limitations of this study. First, the small sample size of forensic psychiatric patients contributes to unstable parameter estimates. Due to WLS estimation being more sample‐size demanding, we were forced to rely on MLR estimation even though the response scale is ordinal in nature. This is because the number of item thresholds to be estimated increases with n*(*k* − 1) with *n* being the number of items and *k* being the number of response categories. It should be noted that since sample size requirements increase (partly) as a function of the number of items and response options, more economical questionnaires can be more rigorously evaluated in smaller sample sizes. Second, DERS‐16 and DERS‐8 were nested within the battery of the DERS‐36 items and not administered independently which would have been favorable as the different DERS versions would have been independent from one another. Notwithstanding these limitations, this study holds several strengths. First, we used two naturalistic forensic samples, enhancing the generalizability and applicability of our findings to clinical settings. On this note, we did not pursue to test measurement invariance due to the large differences in sample size between our two samples although we consider measurement invariance an important feature for future research to explore. Second, we tested convergent validity in at least one sample through examining the relationship between DERS and ERQ. Previous research has to a greater extent relied on criteria of psychiatric symptoms whereas few have investigated the convergence of two different measures of emotion dysregulation. Third, our choice of models was guided by (1) common models used in the literature and (2) clinical challenges within forensic mental health services. Instead of testing a large set of different models and being guided (exclusively) by model fit indices, we aimed at investigating models that either are common in clinical practice or that may hold certain advantages considering their brevity (e.g., DERS‐8 and DERS‐16). This is in line with recommendations within factor analytical research that stress the importance of considering theoretical and clinical implications of the models tested rather than “chasing” optimal fit indices (Sellbom and Tellegen 2019). Lastly, it should be noted that the prison sample was composed of only male individuals and 86% of the forensic psychiatric sample was composed of male subjects. Our findings may not translate equally well to incarcerated women or female forensic psychiatric patients (Revelj et al. 2023).

## 5 Conclusions

To our knowledge, this is the first study to examine the factor structure and psychometric properties of the DERS in forensic samples. Although fit indices yielded divergent results, findings consistently indicated poor data–model fit for the six‐factor correlated traits model. One plausible explanation for this pattern is the weak convergence of the Awareness subscale with the remaining DERS subscales, a finding that aligns with previous research on the DERS. Fit indices improved for the DERS‐16 and DERS‐8 models, although RMSEA values remained elevated. Given the sensitivity of RMSEA to model complexity and degrees of freedom, particularly in larger models, these results should be interpreted in conjunction with other fit indices and evidence of reliability and validity. Taken together, the findings suggest that the DERS‐16 and DERS‐8 may hold promise as measures of emotion dysregulation in forensic clinical settings, although replication in larger samples is warranted. The clinical utility of brief ER measures is particularly relevant in forensic contexts, where assessment burden and lower literacy levels may compromise the feasibility of longer instruments. Furthermore, the present results indicated limited differentiation between DERS subscales in their associations with clinically relevant external correlates. This pattern suggests that, in forensic populations, the composite score may function as a more parsimonious and practically useful index of global emotion dysregulation.

## Author Contributions

Conceptualization: Adam Meddeb, Märta Wallinius, Malin Hildebrand Karlén, and Carlo Garofalo. Data curation: Märta Wallinius, Johan Berlin, Natalie Laporte, Claudia Mazzeschi, Elisa Delvecchio, Patrizia Velotti, and Carlo Garofalo. Methodology: Adam Meddeb, Märta Wallinius, Malin Hildebrand Karlén, and Carlo Garofalo. Formal analysis and investigation: Adam Meddeb. Funding acquisition: Adam Meddeb and Märta Wallinius. Project administration: Adam Meddeb. Supervision: Märta Wallinius, Malin Hildebrand Karlén, and Carlo Garofalo. Writing – original draft preparation: Adam Meddeb. Writing – review and editing: Adam Meddeb, Märta Wallinius, Johan Berlin, Natalie Laporte, Malin Hildebrand Karlén, Claudia Mazzeschi, Elisa Delvecchio, Patrizia Velotti, and Carlo Garofalo.

## Ethics Statement

This study was performed in line with the principles of the Declaration of Helsinki. Approval was granted by the Ethics Committee of Linköping University (2016/213‐31 and 2017/252‐32) and by the Ethics Review Board of Sapienza University of Rome (10/2024).

## Consent

Informed consent was obtained from all individual participants included in the study.

## Conflicts of Interest

The authors declare no conflicts of interest.

## Supporting information


Supporting File

## Data Availability

The data for this study are not available for sharing as this was not permitted by the ethics review authority. All code used to produce the results in this study is publicly and freely available at https://osf.io/xd2fc/overview?view_only=768ea14288c84609bfa31c38bc1787da.
