# Supplementary material for: The Psychometric Properties and Clinical Usefulness of the Difficulties in Emotion Regulation Scale in Forensic Settings
Source: J Clin Psychol. 2026 Jul 23;82(11):1792–808. doi: 10.1002/jclp.70193 (PMC13630352; doi:10.1002/jclp.70193)
Supplement: Supplementary file 1 [file JCLP-82-1792-s001.docx]

**Fig. S1** Distribution of DERS-36, DERS-16 and DERS-8 in the forensic psychiatric sample and in the prison sample.

*Note*. Group 1, forensic psychiatric sample; Group 2, prison sample.
